# Molecular Mechanisms Underlying Activity-Dependent GABAergic Synapse Development and Plasticity and Its Implications for Neurodevelopmental Disorders

**DOI:** 10.1155/2011/734231

**Published:** 2011-08-04

**Authors:** Bidisha Chattopadhyaya

**Affiliations:** Centre de Recherche, CHU Ste-Justine, Université de Montréal, 3175, Chemin de la Côte-Ste-Catherine, Montréal, Q C, Canada H3T 1C5

## Abstract

GABAergic interneurons are critical for the normal function and development of neural circuits, and their dysfunction is implicated in a large number of neurodevelopmental disorders. Experience and activity-dependent mechanisms play an important role in GABAergic circuit development, also recent studies involve a number of molecular players involved in the process. Emphasizing the molecular mechanisms of GABAergic synapse formation, in particular basket cell perisomatic synapses, this paper draws attention to the links between critical period plasticity, GABAergic synapse maturation, and the consequences of its dysfunction on the development of the nervous system.

## 1. Introduction

More than four decades of research has demonstrated that although the brain remains plastic throughout life, continuously reorganizing its connections in the face of new experiences, childhood represents a specific phase in the development of the synaptic network that is characterized by overall remarkable plasticity. During this period of enhanced plasticity also called “critical period”, experience can produce permanent, large-scale changes in neural circuits. Studies on mechanisms that underlie activation and regulation of critical periods in the central nervous system (CNS) are seminal in neuroscience, with the underlying motive being that manipulation of such mechanisms may potentially allow reactivation of neural circuit plasticity during times when the adult brain is less plastic, for example, to aid adaptive circuit rewiring following insult, such as stroke. Additionally, this line of inquiry may help us develop rational pharmacological approaches to correct alterations in the brain of children with neurodevelopmental disorders involving altered synapse formation and/or plasticity.

Critical periods have been observed across sensory, motor, auditory, and also higher cognitive areas; however much of our knowledge of the cellular and molecular mechanisms of onset, maintenance, and termination of these periods derive from seminal studies by Wiesel and Hubel [1] in the developing cat visual system. Electrophysiological recordings from neurons in the primary visual cortex show activation to different degrees by visual stimuli presented to one eye or the other, a property termed ocular dominance. Closing one eye during a specific postnatal time period starts a cascade of events leading to synaptic reorganization of neural circuits in visual cortex, resulting in lifelong, irreversible reduction of the ability of the deprived eye to drive neuronal responses in the cortex, and a dramatic increase in the number of neurons responsive to stimuli presented to the open eye. Such change in eye preference best able to elicit a response from cortical neurons in visual cortex following manipulation of visual inputs is called ocular dominance (OD) plasticity. In marked contrast to what happen in young animals, prolonged eye closure in adults elicits no change in visual cortical neuron responsiveness [2]. Further, monocular deprivation during critical period causes loss of visual acuity in the deprived eye, which is not ameliorated by subsequent experience [3]. This is supported by human studies showing that treatment of amblyopia in children between 7 and 17 years of age was effective only in a fourth of the patients, and to a lesser degree than treatment in younger children [4]. To date, ocular dominance plasticity remains the best-studied experimental model for experience-dependent refinement of neuronal circuits because of the ease of manipulating visual experience independently in the two eyes. 

An important question is which factors determine the timing of critical period plasticity. One of the main players implicated in the onset of critical period plasticity is the development of inhibitory circuitry [5, 6]. Cortical inhibitory neurons, or interneurons, comprise *∼*20–30% of all cortical neurons and predominantly use gamma-aminobutyric acid (GABA) as neurotransmitter. GABAergic interneurons control several aspects of neuronal circuit function from neuronal excitability [7] and integration [8], to the generation of temporal synchrony and oscillation among networks of excitatory neurons [9]. In addition, GABAergic interneurons also regulate key developmental steps, from cell migration and differentiation to experience-dependent refinement of neuronal connections [10, 11]. In the last years, many studies have started to elucidate the development and function of cortical GABAergic circuits.

In this paper the focus is on the molecular mechanisms regulating postnatal GABAergic circuit development and the onset of critical period plasticity, followed by a brief discussion on how aberrations in inhibitory circuit development and alteration in the timing of critical period plasticity could be implicated in neurodevelopmental diseases. 

## 2. GABAergic Inhibition and the Onset of Critical Period

What dictates the time window of a heightened period of plasticity in the brain? Recent studies indicate that the development of inhibitory circuitry in the cortex plays a pivotal role in controlling the onset and time course of critical periods [5, 10, 12]. In particular, two elegant studies envisage a direct role of GABA in the onset of OD plasticity. In a first study, Hensch and collaborators [13] found that mice lacking the synaptic isoform of GABA-producing enzyme, Glutamic Acid Decarboxylase (GAD65), show no OD plasticity. This deficit can be rescued by cortical infusion of the GABAa receptor agonist diazepam, demonstrating that a decrease in inhibition effectively abolished critical period and impaired plasticity mechanisms. In the second study, Fagiolini and Hensch [5] showed that the early enhancement of GABA-mediated inhibition by diazepam application triggers the precocious onset of OD plasticity. Further, precocious development of inhibitory circuitry via action of the Brain Derived Neurotrophic Factor (BDNF) accelerates the onset of the critical period for visual plasticity [12].

Cortical GABAergic interneurons form a strikingly diverse and heterogenous group differing in morphology, physiological properties, and protein expression [14, 15]. The hypothesis that different interneuron subtypes play different roles in cortical development, function, and plasticity is therefore a tantalizing one. Fagiolini et al. [16] showed that GABA transmission mediated by the *α*1 subunit-containing GABAa receptors is required for the induction of critical period for OD plasticity. Because different classes of inhibitory synapses preferentially signal through GABAa receptors with different subunit composition [17], these results suggest that maturation of specific subclasses of GABA interneurons is crucial to initiate critical period plasticity. More recent data indicate that site-specific optimization of GABAa receptor numbers on the soma-proximal dendritic compartment of pyramidal cells triggers the onset of OD plasticity [18]. The soma proximal dendritic compartment of pyramidal cells is preferentially innervated by Parvalbumin (Pv) positive basket interneurons. Taken altogether, these data suggest a critical role for basket cell interneuron maturation in the onset of critical period plasticity.

A novel mechanism explaining how visual input is coupled to the onset of ocular dominance plasticity has been proposed by Sugiyama et al. [19]. Traditionally, the molecular signals linking visual experience to GABA interneuron maturation were thought to be recruited from within the cortex itself, such as the activity-dependent synthesis and release of BDNF by pyramidal neurons [12]. Instead, Sugiyama et al. [19] demonstrated that a retina-derived homeoprotein, Otx2, is first transferred into the primary visual cortex via a visual experience-dependent mechanism. Once in the cortex, Otx2 then nurtures GABAergic interneurons and promotes critical period plasticity. The investigation of the target genes and proteins of Otx2 will reveal further insights into the mechanisms linking experience, GABAergic circuit maturation, and critical period plasticity. 

## 3. Molecular Mechanisms of GABAergic Circuit Development

The GABAergic network comprises of diverse interneuron subtypes that have different morphological and physiological characteristics and localize their synapses onto distinct subcellular locations on the postsynaptic targets. Precisely how activity and molecular-driven mechanisms conspire to achieve the remarkable specificity of GABAergic synapse localization and formation is unknown. The functional maturation of GABA-mediated inhibition is a prolonged process that extends well into adolescence, both in rodents and primates [20–23], and correlates with the time course of the critical period for OD plasticity [21, 23]. Moreover, the inhibitory maturation process strongly depends on sensory experience, since sensory deprivation, induced either by dark rearing or by intraocular tetradotoxin (TTX) injection, significantly retards the morphological and functional maturation of GABAergic synapses [21, 23]. This dependence of GABAergic synapse maturation on sensory experience is not limited to visual cortex, indeed similar results have been found in the somatosensory cortex [24]. 

What are the cellular and molecular mechanisms linking sensory experience to the maturation of GABAergic synapses? Brain-Derived Neurotrophic Factor (BDNF), an activity-dependent molecule shown to be upregulated following light stimulation in the visual cortex [25, 26], is one of the first molecules implicated in the formation of GABAergic synapses in hippocampal and cortical cultures [27, 28]. Most importantly, in transgenic mice with precocious BDNF expression, a marked increase in perisomatic inhibitory innervation in the visual cortex is correlated with a premature onset and closure of ocular dominance plasticity, further supporting the link between GABAergic synapse maturation and onset of critical period plasticity [12, 29]. Since BDNF is produced only by pyramidal cells, it could work as an intercellular signaling factor that translates pyramidal cell activity to GABAergic synapse density.

**Figure 1:**
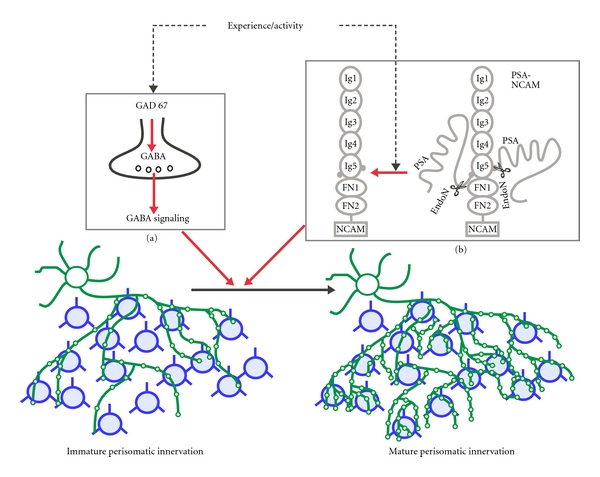
Sensory activity regulates perisomatic synapse maturation via multiple pathways. (a) Activity modulates GAD67 enzyme levels thereby ensuring normal GABA signaling for the appropriate downstream signaling events required for perisomatic synapse development. (b) Experience is also critical for removal of the PSA moiety from NCAM, allowing onset of perisomatic innervation at the right time.

Another factor that has been shown to positively regulate GABAergic synapse maturation is GABA itself. Early in development, GABA has been shown to be a trophic factor [30], involved in cell proliferation, neuronal migration, and neurite growth [31]. Since GAD67 is the main isoform of GABA synthesizing enzyme, its deletion reduces GABA levels by 90% [32]. Using transgenic mice to knockdown GAD67 in single basket interneurons during the period of their maturation, recent studies show that intact GABA signaling is critical for the maturation of GABAergic synapses [33] (Figure 1). Intriguingly, even a partial reduction of GAD67 was sufficient to cause aberrant perisomatic synapse maturation, underlying the importance of maintaining optimal GABA levels for normal synapse development [33]. Basket cell perisomatic synapses have an exuberant innervation pattern; a single basket interneuron connects to hundreds of pyramidal cells in its vicinity, making numerous synapses onto each individual pyramidal cell soma. It is therefore important to appreciate that reduced GABA levels compromise not only the number of synapses that are made onto each pyramidal soma, but also drastically reduce the number of pyramidal soma it connects to, causing a potential circuit-wide disruption in connectivity [33]. This study demonstrates that, in addition to mediating inhibitory transmission, GABA signaling also regulates interneuron axon arborization and synapse development in adolescent brain, which, in turn regulates critical period plasticity. Different aspects of this deficit were rescued by treatment with either GABAa or GABAb agonists, suggesting a receptor-specific effect of GABA-mediated signaling during GABAergic synapse maturation [33]. Since GABAa and GABAb receptors are present on postsynaptic neurons, GABA terminal themselves, and surrounding glial processes, cell-autonomous activation of presynaptic GABAb receptors, which modulate Ca^2+^channels and GABA release, could influence growth cone motility and bouton stability, or GABA signaling through postsynaptic or glia receptors could trigger the release of retrograde factors, which promote axon branching and synapse formation. 

Modulation of GABA synthesis by the GAD67 enzyme plays a central role in regulating GABA-mediated signaling [34]. GAD67 itself is produced at a limiting level in the brain, since deletion of one copy of the *Gad1* gene results in a *∼*40% reduction of enzyme activity and GABA content in many brain regions [32]. Furthermore, the transcription of *Gad1*, the key step in the physiological control of GAD67 activity, is highly regulated during brain development [35], by neuronal activity [36], and experience [37, 38]. Activity-dependent production of GAD67 thus results in online adjustment of intracellular pool for GABA release. Since alterations in GAD67 and GABA levels profoundly influence interneuron axon growth, synapse formation and network connectivity during the establishment of inhibitory circuits, neuronal activity might regulate the strength and pattern of inhibitory synaptic innervation through GAD67-mediated GABA synthesis and signaling. Such activity-dependent and cell-wide regulation of a “transmitter resource” implies a novel logic for the maturation and plasticity of GABAergic synapses and innervation. Since subtle variations in GABA levels can cause such dramatic effects on inhibitory circuits, and therefore overall network connectivity, it is critical to understand its implications in neuropsychiatric disorders and strive to regulate optimal GABA levels for proper circuit function. 

A recent study by Fiorentino et al. [39] proposes that the interaction between BDNF and GABA signaling influences GABAergic synapse maturation. The authors demonstrate that activation of metabotropic GABAb receptor triggers secretion of BDNF and promotes the development of GABAergic synapses, in particular, the perisomatic GABAergic synapses, onto CA3 pyramidal neurons in the hippocampus of newborn mice [39]. Whether a similar mechanism is at play in the visual cortex is still unknown; however, the picture so far indicates a positive interplay between sensory experience, BDNF, and GABA signaling, to induce GABAergic synapse maturation and in turn promote the onset of ocular dominance plasticity. 

In addition to factors promoting GABAergic synapse maturation, recent studies have revealed inhibitory mechanisms that set the appropriate time course for establishment of mature GABAergic innervation patterns and the onset of critical period plasticity. In particular, polysialic acid (PSA), linked to the neural cell adhesion molecule (NCAM), acts as a negative signal to suppress the formation of inhibitory synapses and the onset of OD plasticity in the developing visual cortex [40]. In the mammalian brain, NCAM is a predominant carrier of the unusual long-chain, polyanionic carbohydrate, PSA, although outside the nervous system more carriers of PSA are known, including neuropilin-2 [41]. PSA is a long linear homopolymer of *α*-2,8-linked sialic acid that is synthesized in the Golgi by two polysialyltransferases, PST (also known as ST8SiaIV) and STX (also known as ST8SiaII), either of which is sufficient for the complete synthesis of PSA chain on a standard asparaginyl-linked core carbohydrate attached to NCAM [42, 43]. 

One of the most studied characteristics of PSA is its ability to act as a de-adhesive factor, causing steric hindrance, between cellular membranes. Cell surface expression of PSA constricts intercellular space between apposing cells [44], which in turn, decreases homophilic binding between NCAM and other cells adhesion molecules including Cadherins, L1 family, and Integrins [45], therefore acting as a permissive regulating factor rather than a specific instructive cue. PSA affects distinct developmental processes depending on the location and timing of its expression. For example, in the developing nervous system PSA creates conditions permissive for postmitotic migration of precursor cells. In the adult, migrating cells still retain PSA, such as progenitor cells migrating along rostral migratory stream from the subventricular zone to the olfactory bulb [46] and newborn granule cells in the hippocampus [47]. 

Recent studies show the ability of PSA to regulate ocular dominance plasticity [40]. Although PSA expression is highest in the embryonic stages, it is expressed in the postnatal brain at different levels depending on brain region and age. In the mouse visual cortex, PSA expression declines to almost undetectable levels shortly after eye opening, and this decline is attenuated by visual deprivation [40]. Indeed, PSA levels in visual cortex were higher in mice dark reared from birth compared to littermates reared in a normal light-dark cycle. This effect is echoed in the visual cortex contralateral to the eye that received daily intraocular injection of TTX compared to the ipsilateral cortex [40]. Since the developmental and activity-regulated expression of PSA inversely correlates with the maturation of GABAergic innervation [21], it is thus possible that PSA decline might be sufficient for GABAergic synapse maturation. Indeed, premature enzymatic removal of PSA in the developing visual cortex results in precocious maturation of perisomatic innervation by basket interneurons and enhanced inhibitory synaptic transmission. Most importantly, the same treatment causes an earlier onset of critical period plasticity in the visual cortex [40]. Since PSA removal promotes GABAergic synapse formation, and GABA signaling in turn further promotes the maturation of GABAergic innervation [33], together GABA signaling and PSA removal may constitute a positive feedback mechanism to accelerate GABAergic synapse formation once sensory experience begins, and consequently to induce the onset of critical period plasticity in the visual cortex. PSA also regulates glutamatergic synapse formation [48, 49] and affects neuron-glia interactions [50] thus the possibility of additional mechanisms by which PSA influences ocular dominance plasticity cannot be excluded. 

What is the precise role of PSA in GABAergic circuit maturation? One possibility is that developmental and activity-dependent removal of PSA might coordinate the timing of axon and synapse morphogenesis during the maturation of GABAergic innervation; indeed precocious perisomatic synapse formation can be triggered by premature removal of PSA. Excessive, premature synapse formation might constrain axon growth. Higher expression of PSA during the early postnatal weeks might attenuate interactions between basket cell axons and pyramidal neurons, thereby holding off synapse formation and promoting the elaboration of axon arbors. Subsequent activity-dependent removal of PSA might unmask mechanisms that are already in place along basket cell axon, allowing fast responses to local synaptogenic cues. A similar example of PSA regulating the timing of a biological process comes from studies of migrating neuronal precursor. When PSA is enzymatically removed from newly generated cells in the SVZ, they form neuronal processes and begin to express neuronal molecular markers. This premature developmental transition is dependent on cell contact and appears to involve signaling through NCAM and p59Fyn kinase [51]. 

Why is such a mechanism in place and what could be its purpose? Interestingly, long polymers of sialic acid are not found in invertebrates [43], where neural circuits are to a large extent genetically determined. This raises the possibility that PSA might have evolved to regulate vertebrate-specific developmental processes. An example is the role of PSA in cell migration and differentiation. In invertebrates, the differentiation of neuronal precursors occurs close to the region of their birth and involves interactions with its immediate neighbor cells. On the other hand, in vertebrates, newly generated precursors often migrate long distances before acquiring their fate, and thus need to delay their differentiation till they reach their destination. Here, PSA plays a dual role whereby it (a) promotes cell migration by reducing cell-cell adhesion and (b) blocks differentiation by interfering with contact-dependent signaling until the cells arrive at their final location.

Such multifaceted roles for PSA are well suited for the complex experience-dependent neural circuit fine-tuning that occurs in vertebrate CNS. It is interesting to note that vision-dependent critical period plasticity does not start at the onset of eye opening. Instead, it is hypothesized that the critical period cannot start until the input to the circuit has developed reliability and precision [52]. Thus, cellular mechanisms underlying critical period are not simply an activity-dependent process; instead, it is a sequence of timed events that appear to be important. PSA might then act as “brake” that holds off the onset of critical period plasticity until input information can be reliably relayed to the cortex. The challenge is to understand what happens if and when this timing is altered, whether onset of critical period before the appropriate time might lead to incorrect refinement of neural circuit based on unreliable, or nonoptimal inputs, and whether and how this would in turn affect behavior. 

## 4. Implications for Neurodevelopmental Disorders

GABAergic circuit dysfunction has been implicated in various neurodevelopmental and psychiatric disorders such as autism and schizophrenia [22, 53, 54]. Therefore, our understanding of the mechanisms that control formation and plasticity of GABAergic circuits will likely yield molecular and cellular substrates that might be altered in neurodevelopmental disorders.

Efforts to explore molecular mechanisms linking sensory experience to GABAergic circuit maturation have revealed several players that include both GABAergic synapse promoting factors (BDNF, Otx2, and GABA itself) and GABAergic synapse inhibiting factors (PSA). It has become increasingly clear that mechanisms are in place to tightly time events leading to the onset of critical period plasticity. This raises the question as to what maybe the correct or most permissible sequence of events and whether the onset of critical period at a time when circuits are not “ready” could lead to an altered developmental trajectory. 

GABA synthesis and signaling has been shown to regulate the maturation of GABAergic innervation in visual cortex and the onset of critical period plasticity [5, 33]. These findings suggest that alteration of GABA synthesis and signalling, either due to genetic or environmental causes, can potentially affect nearly all stages of cortical circuit formation, thereby leading to impaired brain development. For instance, SNPs in the 5′ regulatory region of the *Gad1* gene (coding for the GABA- synthesizing enzyme GAD67) are associated with childhood onset schizophrenia [55]. Moreover, allelic variations in *Gad1* have been shown to associate with schizophrenia and to influence multiple domains of cognition, including declarative memory, attention and working memory [56]. This is interesting because reduction in the expression levels of GAD67 in the dorsal lateral prefrontal cortex is one of the most consistent molecular pathological findings in individuals with schizophrenia [22]. However, whether and how these genetic variants are directly involved in the regulation of *Gad1* expression levels is still unknown. 

In addition, the multifaceted role of GABA during cortical circuits development draws our attention to the possible deleterious effects of drugs acting on GABA receptors, notably benzodiazepines or certain antiepileptic agents, on brain development. Recent evidence from both clinical and animal studies suggests that certain antiepileptic drugs could interfere with normal cognitive development [57]. Further studies are required to understand if GABA-targeting drugs could have long-term consequences in young children by interfering, between other things, with critical period plasticity. 

GABAergic circuit dysfunction has also been implicated in autism spectrum disorders, including Rett's syndrome [53, 54]. The homeodomain transcription factor Dlx5, which regulates the differentiation and maturation of forebrain GABAergic interneurons, has been identified as a direct target of MeCP2 [58], which is linked to Rett's syndrome. Critical period OD plasticity is altered in MeCP2 mutant mice, a well-recognized model for Rett's syndrome [59]. Recent studies using transgenic mice lacking MeCP2 selectively in GABAergic neurons show that these mice behaviorally recapitulate many features of Rett's syndrome, linking decreased Gad levels and compromised MeCP2 function in GABAergic neurons to the neuropsychiatric phenotype [60].

Altered PSA levels are associated with various neuropathological conditions including schizophrenia [61, 62] and temporal lobe epilepsy [63]. In particular, a decrease in polysialylation of hippocampal neurons in schizophrenic brains correlates with early disease incidence [61, 64]. Recently, the chromosome where ST8SIA2, the human STX-encoding gene, is localized, 15q26, was reported as a common susceptibility region for both schizophrenia and bipolar disorder in a genome scan of Eastern Quebec families [65]. Convergent evidence from the Chinese Han and Japanese population [66, 67] strongly supports the possibility that developmental abnormalities associated with defective polysialylation may be involved in schizophrenia.

In summary, multiple lines of evidence concur that alterations in molecular mechanisms of GABAergic synapse development and regulation of critical period plasticity are associated with neurodevelopmental disorders. Aberrant development of GABAergic circuits has been implicated in various dysfunctions such as autism, schizophrenia, Rett syndrome, and epilepsy. Further research along these lines will help elucidate how and whether critical period plasticity is affected, which molecular pathway is critical, and whether therapeutic intervention is possible. Exciting recent evidence points to possible strategies to reopen plasticity in a mature brain [68–70]. Altogether, increasing knowledge of such molecular mechanisms will further our understanding of the regulation of developmental plasticity in the brain and aid in designing strategies aimed to increase adaptive circuit rewiring following insult, such as stroke, and in developing rational pharmacological approaches to correct alterations in the brain of children with neurodevelopmental disorders. 

## References

[1] Wiesel TN, Hubel DH (1963). Single-cell responses in striate cortex of kittens deprived of vision in one eye. *Journal of Neurophysiology*.

[2] Hubel DH, Wiesel TN (1970). The period of susceptibility to the physiological effects of unilateral eye closure in kittens. *Journal of Physiology*.

[3] Berardi N, Pizzorusso T, Maffei L (2000). Critical periods during sensory development. *Current Opinion in Neurobiology*.

[4] Scheiman MM, Hertle RW, Beck RW (2005). Randomized trial of treatment of amblyopia in children aged 7 to 17 years. *Archives of Ophthalmology*.

[5] Fagiolini M, Hensch TK (2000). Inhibitory threshold for critical-period activation in primary visual cortex. *Nature*.

[6] Huang ZJ, Di Cristo G, Ango F (2007). Development of GABA innervation in the cerebral and cerebellar cortices. *Nature Reviews Neuroscience*.

[7] Swadlow HA (2003). Fast-spike interneurons and feedforward inhibition in awake sensory neocortex. *Cerebral Cortex*.

[8] Pouille F, Scanziani M (2001). Enforcement of temporal fidelity in pyramidal cells by somatic feed-forward inhibition. *Science*.

[9] Somogyi P, Klausberger T (2005). Defined types of cortical interneurone structure space and spike timing in the hippocampus. *Journal of Physiology*.

[10] Hensch TK, Fagiolini M (2005). Excitatory-inhibitory balance and critical period plasticity in developing visual cortex. *Progress in Brain Research*.

[11] Ben-Ari Y (2002). Excitatory actions of GABA during development: the nature of the nurture. *Nature Reviews Neuroscience*.

[12] Huang ZJ, Kirkwood A, Pizzorusso T (1999). BDNF regulates the maturation of inhibition and the critical period of plasticity in mouse visual cortex. *Cell*.

[13] Hensch TK, Fagiolini M, Mataga N, Stryker MP, Baekkeskov S, Kash SF (1998). Local GABA circuit control of experience-dependent plasticity in developing visual cortex. *Science*.

[14] Kawaguchi Y, Kubota Y (1997). GABAergic cell subtypes and their synaptic connections in rat frontal cortex. *Cerebral Cortex*.

[15] Markram H, Toledo-Rodriguez M, Wang Y, Gupta A, Silberberg G, Wu C (2004). Interneurons of the neocortical inhibitory system. *Nature Reviews Neuroscience*.

[16] Fagiolini M, Fritschy JM, Löw K, Möhler H, Rudolph U, Hensch TK (2004). Specific GABA_A_ circuits for visual cortical plasticity. *Science*.

[17] Ali AB, Thomson AM (2008). Synaptic *α*5 subunit-containing GABA_A_ receptors mediate IPSPs elicited by dendrite-preferring cells in rat neocortex. *Cerebral Cortex*.

[18] Katagiri H, Fagiolini M, Hensch TK (2007). Optimization of somatic inhibition at critical period onset in mouse visual cortex. *Neuron*.

[19] Sugiyama S, Di Nardo AA, Aizawa S (2008). Experience-dependent transfer of Otx2 homeoprotein into the visual cortex activates postnatal plasticity. *Cell*.

[20] Luhmann HJ, Prince DA (1991). Postnatal maturation of the GABAergic system in rat neocortex. *Journal of Neurophysiology*.

[21] Chattopadhyaya B, Di Cristo G, Higashiyama H (2004). Experience and activity-dependent maturation of perisomatic GABAergic innervation in primary visual cortex during a postnatal critical period. *The Journal of Neuroscience*.

[22] Lewis DA, Hashimoto T, Volk DW (2005). Cortical inhibitory neurons and schizophrenia. *Nature Reviews Neuroscience*.

[23] Morales B, Choi SY, Kirkwood A (2002). Dark rearing alters the development of GABAergic transmission in visual cortex. *The Journal of Neuroscience*.

[24] Jiao Y, Zhang C, Yanagawa Y, Sun QQ (2006). Major effects of sensory experiences on the neocortical inhibitory circuits. *The Journal of Neuroscience*.

[25] Bozzi Y, Pizzorusso T, Cremisi F, Rossi FM, Barsacchi G, Maffei L (1995). Monocular deprivation decreases the expression of messenger RNA for brain-derived neurotrophic factor in the rat visual cortex. *Neuroscience*.

[26] Castren E, Zafra F, Thoenen H, Lindholm D (1992). Light regulates expression of brain-derived neurotrophic factor mRNA in rat visual cortex. *Proceedings of the National Academy of Sciences of the United States of America*.

[27] Vicario-Abejón C, Collin C, McKay RDG, Segal M (1998). Neurotrophins induce formation of functional excitatory and inhibitory synapses between cultured hippocampal neurons. *The Journal of Neuroscience*.

[28] Rutherford LC, DeWan A, Lauer HM, Turrigiano GG (1997). Brain-derived neurotrophic factor mediates the activity-dependent regulation of inhibition in neocortical cultures. *The Journal of Neuroscience*.

[29] Hanover JL, Huang ZJ, Tonegawa S, Stryker MP (1999). Brain-derived neurotrophic factor overexpression induces precocious critical period in mouse visual cortex. *The Journal of Neuroscience*.

[30] Represa A, Ben-Ari Y (2005). Trophic actions of GABA on neuronal development. *Trends in Neurosciences*.

[31] Owens DF, Kriegstein AR (2002). Is there more to GABA than synaptic inhibition?. *Nature Reviews Neuroscience*.

[32] Asada H, Kawamura Y, Maruyama K (1997). Cleft palate and decreased brain *γ*-aminobutyric acid in mice lacking the 67-kDa isoform of glutamic acid decarboxylase. *Proceedings of the National Academy of Sciences of the United States of America*.

[33] Chattopadhyaya B, Di Cristo G, Wu CZ (2007). GAD67-mediated GABA synthesis and signaling regulate inhibitory synaptic innervation in the visual cortex. *Neuron*.

[34] Pinal CS, Tobin AJ (1998). Uniqueness and redundancy in GABA production. *Perspectives on Developmental Neurobiology*.

[35] Kiser PJ, Cooper NG, Mower GD (1998). Expression of two forms of glutamic acid decarboxylase (GAD67 and GAD65) during postnatal development of rat somatosensory barrel cortex. *The Journal of Comparative Neurology*.

[36] Patz S, Wirth MJ, Gorba T, Klostermann O, Wahle P (2003). Neuronal activity and neurotrophic factors regulate GAD-65/67 mRNA and protein expression in organotypic cultures of rat visual cortex. *European Journal of Neuroscience*.

[37] Benevento LA, Bakkum BW, Cohen RS (1995). Gamma-aminobutyric acid and somatostatin immunoreactivity in the visual cortex of normal and dark-reared rats. *Brain Research*.

[38] Benson DL, Isackson PJ, Hendry SHC, Jones EG (1989). Expression of glutamic acid decarboxylase mRNA in normal and monocularly deprived cat visual cortex. *Molecular Brain Research*.

[39] Fiorentino H, Kuczewski N, Diabira D (2009). GABA_B_ receptor activation triggers BDNF release and promotes the maturation of GABAergic synapses. *The Journal of Neuroscience*.

[40] Di Cristo G, Chattopadhyaya B, Kuhlman SJ (2007). Activity-dependent PSA expression regulates inhibitory maturation and onset of critical period plasticity. *Nature Neuroscience*.

[41] Curreli S, Arany Z, Gerardy-Schahn R, Mann D, Stamatos NM (2007). Polysialylated neuropilin-2 is expressed on the surface of human dendritic cells and modulates dendritic cell-T lymphocyte interactions. *The Journal of Biological Chemistry*.

[42] Angata K, Fukuda M (2003). Polysialyltransferases: major players in polysialic acid synthesis on the neural cell adhesion molecule. *Biochimie*.

[43] Rutishauser U (2008). Polysialic acid in the plasticity of the developing and adult vertebrate nervous system. *Nature Reviews Neuroscience*.

[44] Johnson CP, Fujimoto I, Rutishauser U, Leckband DE (2005). Direct evidence that Neural Cell Adhesion Molecule (NCAM) polysialylation increases intermembrane repulsion and abrogates adhesion. *The Journal of Biological Chemistry*.

[45] Fujimoto I, Bruses JL, Rutishauser U (2001). Regulation of cell adhesion by polysialic acid: effects on cadherin, immunoglobulin cell adhesion molecule, and integrin function and independence from neural cell adhesion molecule binding or signaling activity. *The Journal of Biological Chemistry*.

[46] Hu H, Tomasiewicz H, Magnuson T, Rutishauser U (1996). The role of polysialic acid in migration of olfactory bulb interneuron precursors in the subventricular zone. *Neuron*.

[47] Burgess A, Wainwright SR, Shihabuddin LS, Rutishauser U, Seki T, Aubert I (2008). Polysialic acid regulates the clustering, migration, and neuronal differentiation of progenitor cells in the adult hippocampus. *Developmental Neurobiology*.

[48] Dityatev A, Dityateva G, Sytnyk V (2004). Polysialylated neural cell adhesion molecule promotes remodeling and formation of hippocampal synapses. *The Journal of Neuroscience*.

[49] Muller D, Wang C, Skibo G (1996). PSA-NCAM is required for activity-induced synaptic plasticity. *Neuron*.

[50] Theodosis DT, Bonhomme R, Vitiello S, Rougon G, Poulain DA (1999). Cell surface expression of polysialic acid on NCAM is a prerequisite for activity-dependent morphological neuronal and glial plasticity. *The Journal of Neuroscience*.

[51] Petridis AK, El Maarouf A, Rutishauser U (2004). Polysialic acid regulates cell contact-dependent neuronal differentiation of progenitor cells from the subventricular zone. *Developmental Dynamics*.

[52] Knudsen EI (2004). Sensitive periods in the development of the brain and behavior. *Journal of Cognitive Neuroscience*.

[53] Dani VS, Chang Q, Maffei A, Turrigiano GG, Jaenisch R, Nelson SB (2005). Reduced cortical activity due to a shift in the balance between excitation and inhibition in a mouse model of Rett syndrome. *Proceedings of the National Academy of Sciences of the United States of America*.

[54] Belmonte MK, Cook EH, Anderson GM (2004). Autism as a disorder of neural information processing: directions for research and targets for therapy. *Molecular Psychiatry*.

[55] Addington AM, Gornick M, Duckworth J (2005). *GAD1* (2q31.1), which encodes glutamic acid decarboxylase (GAD_67_), is associated with childhood-onset schizophrenia and cortical gray matter volume loss. *Molecular Psychiatry*.

[56] Straub RE, Lipska BK, Egan MF (2007). Allelic variation in GAD1 (GAD_67_) is associated with schizophrenia and influences cortical function and gene expression. *Molecular Psychiatry*.

[57] Marsh ED, Brooks-Kayal AR, Porter BE (2006). Seizures and antiepileptic drugs: does exposure alter normal brain development?. *Epilepsia*.

[58] Horike SI, Cai S, Miyano M, Cheng JF, Kohwi-Shigematsu T (2005). Loss of silent-chromatin looping and impaired imprinting of DLX5 in Rett syndrome. *Nature Genetics*.

[59] Tropea D, Giacometti E, Wilson NR (2009). Partial reversal of Rett syndrome-like symptoms in MeCP2 mutant mice. *Proceedings of the National Academy of Sciences of the United States of America*.

[60] Chao HT, Chen H, Samaco RC (2010). Dysfunction in GABA signalling mediates autism-like stereotypies and Rett syndrome phenotypes. *Nature*.

[61] Barbeau D, Liang JJ, Robitaille Y, Quirion R, Srivastava LK (1995). Decreased expression of the embryonic form of the neural cell adhesion molecule in schizophrenic brains. *Proceedings of the National Academy of Sciences of the United States of America*.

[62] Isomura R, Kitajima K, C. Sato (2011). Structural and functional impairments of polysialic acid by a mutated polysialyltransferase found in schizophrenia. *The Journal of Biological Chemistry*.

[63] Mikkonen M, Soininen H, Kälviäinen R (1998). Remodeling of neuronal circuitries in human temporal lobe epilepsy: increased expression of highly polysialylated neural cell adhesion molecule in the hippocampus and the entorhinal cortex. *Annals of Neurology*.

[64] Vicente AM, Macciardi F, Verga M (1997). NCAM and schizophrenia: genetic studies. *Molecular Psychiatry*.

[65] Maziade M, Roy MA, Chagnon YC (2005). Shared and specific susceptibility loci for schizophrenia and bipolar disorder: a dense genome scan in Eastern Quebec families. *Molecular Psychiatry*.

[66] Arai M, Yamada K, Toyota T (2006). Association between polymorphisms in the promoter region of the sialyltransferase 8B (SIAT8B) gene and schizophrenia. *Biological Psychiatry*.

[67] Tao R, Li C, Zheng Y (2007). Positive association between SIAT8B and schizophrenia in the Chinese Han population. *Schizophrenia Research*.

[68] Pizzorusso T, Medini P, Berardi N, Chierzi S, Fawcett JW, Maffei L (2002). Reactivation of ocular dominance plasticity in the adult visual cortex. *Science*.

[69] Sale A, Maya Vetencourt JF, Medini P (2007). Environmental enrichment in adulthood promotes amblyopia recovery through a reduction of intracortical inhibition. *Nature Neuroscience*.

[70] Vetencourt JFM, Sale A, Viegi A (2008). The antidepressant fluoxetine restores plasticity in the adult visual cortex. *Science*.

